# Supplementary material for: The major β-catenin/E-cadherin junctional binding site is a primary molecular mechano-transductor of differentiation in vivo
Source: eLife. 2018 Jul 19;7:e33381. doi: 10.7554/eLife.33381 (PMC6053302; doi:10.7554/eLife.33381)
Supplement: Figure 1—source data 1. [file elife-33381-fig1-data1.docx]

**F=0pN, 30ns**

Distance end-to-end Y142-β-cat-A648-Ecad (Å) :

108.8, 116.3, 111.7, 111.2, 110.1, 111.3, 109.7, 110.0, 106.8, 106.3

Distance C-terminus-β-cat-A648-Ecad  FRET (Å) :

16.0, 17.8, 16.2, 15.4, 17.2, 16.4, 15.8, 17.8, 18.3, 17.8

Distance Y654-D665 (Å) :

5.3, 5.6, 5.2, 5.5, 5.5, 5.3, 5.2, 5.3, 4.2, 5.6

**F=150pN, 30ns**

Distance end-to-end Y142-β-cat-A648-Ecad (Å) [15-30] ns :

186.8, 188.2, 191.9, 183.6, 190.3, 186.2, 200.2, 187.8, 178.3, 197.7, 197.6, 201.4, 184.9, 183.5, 194.5, 183.0, 184.2, 176.7, 183.5, 172.9

Distance C-terminus-β-cat-A648-Ecad  FRET (Å) [15-30] ns:

23.4, 25.6, 29.8, 25.2, 21.4, 24.9, 24.5, 32.3, 32.8, 42.4, 33.3, 24.2, 25.6, 26.3, 24.9, 24.3, 24.8, 26.4, 23.1, 18.8

Distance Y654-D665 (Å) [15-30] ns:

5.3, 4.4, 5.5, 5.2, 5.3, 5.4, 4.4, 5.5, 9.2, 10.4, 7.2, 3.8, 5.3, 3.9, 4.7, 4.2, 3.7, 4.0, 5.2, 5.5
