# Supplementary material for: The major β-catenin/E-cadherin junctional binding site is a primary molecular mechano-transductor of differentiation in vivo
Source: eLife. 2018 Jul 19;7:e33381. doi: 10.7554/eLife.33381 (PMC6053302; doi:10.7554/eLife.33381)
Supplement: Figure 2—source data 1. [file elife-33381-fig2-data1.txt]

Lifetime								
	Antibody					PBS control		
	mesoderm	ectoderm	mesoderm	ectoderm		mesoderm	mesoderm	
	stage 5	stage 5	stage6	stage6		stage5	stage6	
	2.342	2.502	2.545	2.468		2.634	2.639	
	2.458	2.409	2.691	2.568		2.528	2.648	
	2.453	2.454	2.535	2.545		2.603	2.651	
	2.562	2.565	2.491	2.416		2.626	2.611	
	2.458	2.588	2.598	2.361		2.581	2.595	
	2.351	2.505	2.613	2.457		2.656	2.511	
	2.515	2.453	2.54	2.463		2.561	2.613	
	2.554	2.557	2.492	2.567		2.597	2.551	
	2.46		2.567	2.546		2.611	2.618	
	2.528		2.618	2.544				
	2.517		2.542					
								
								
								
Average	2.473	2.504	2.567	2.494		2.600	2.604	
STDEV	0.074	0.063	0.059	0.071		0.039	0.046	
SEM	0.022	0.022	0.018	0.022		0.013	0.015	
Differences to mean stage 5 mesoderm								
	Antibody					PBS control		
	mesoderm	ectoderm	mesoderm	ectoderm		mesoderm	mesoderm	
	stage 5	stage 5	stage6	stage6		stage5	stage6	
	-0.131	0.029	0.072	-0.005		0.034	0.039	
	-0.015	-0.064	0.218	0.095		-0.072	0.048	
	-0.020	-0.019	0.062	0.072		0.003	0.051	
	0.089	0.092	0.018	-0.057		0.026	0.011	
	-0.015	0.115	0.125	-0.112		-0.019	-0.005	
	-0.122	0.032	0.140	-0.016		0.056	-0.089	
	0.042	-0.020	0.067	-0.010		-0.039	0.013	
	0.081	0.084	0.019	0.094		-0.003	-0.049	
	-0.013		0.094	0.073		0.011	0.018	
	0.055		0.145	0.071				
	0.044		0.069					
								
								
								
Average	0.000	0.032	0.094	0.021		0.000	0.004	
STDEV	0.074	0.063	0.059	0.071		0.039	0.046	
SEM	0.022	0.022	0.018	0.022		0.013	0.015	
