# Supplementary material for: The major β-catenin/E-cadherin junctional binding site is a primary molecular mechano-transductor of differentiation in vivo
Source: eLife. 2018 Jul 19;7:e33381. doi: 10.7554/eLife.33381 (PMC6053302; doi:10.7554/eLife.33381)
Supplement: Figure 2—source data 2. [file elife-33381-fig2-data2.txt]

Stage 5	Stage 6	Stage 7	
2.640	2.611	2.599	
2.642	2.607	2.646	
2.632	2.604	2.652	
2.613	2.617	2.572	
2.609	2.617	2.578	
2.541	2.617	2.567	
2.551	2.621	2.549	
2.514	2.617		
2.510	2.614		
2.521	2.613		
2.615	2.604		
2.640	2.577		
	2.574		
	2.577		
