# Supplementary material for: The major β-catenin/E-cadherin junctional binding site is a primary molecular mechano-transductor of differentiation in vivo
Source: eLife. 2018 Jul 19;7:e33381. doi: 10.7554/eLife.33381 (PMC6053302; doi:10.7554/eLife.33381)
Supplement: Figure 3—source data 1. [file elife-33381-fig3-data1.txt]

Antibody Magnet				
	before	during	difference	
	2.585	2.660	0.075	
	2.567	2.726	0.159	
	2.577	2.635	0.058	
	2.578	2.767	0.189	
	2.568	2.601	0.033	
	2.516	2.599	0.083	
	2.516	2.664	0.148	
	2.502	2.529	0.027	
	2.533	2.641	0.108	
				
				
Mean	2.549	2.647	0.098	
STDEV	0.032110919	0.070376566	0.057251152	
SEM	0.901246876	0.935816541	0.034569665	
Antibody No Magnet				
	before	during		
	2.586	2.629	0.043	
	2.573	2.559	-0.014	
	2.657	2.648	-0.009	
	2.506	2.523	0.017	
	2.48	2.454	-0.026	
	2.576	2.578	0.002	
	2.542	2.561	0.019	
	2.519	2.475	-0.044	
				
				
Mean	2.555	2.553	-0.001	
STDEV	0.055461027	0.067962079	0.027784128	
SEM	0.903271461	0.90276912	-0.00050234	
PBS Magnet				
	before	during	difference	
	2.69	2.647	-0.043	
	2.6	2.591	-0.009	
	2.654	2.626	-0.028	
	2.629	2.651	0.022	
	2.632	2.665	0.033	
	2.594	2.579	-0.015	
				
				
				
				
				
Mean	2.633	2.627	-0.007	
STDEV	0.03554387	0.034697262	0.029138749	
SEM	0.995243452	0.992723688	-0.002519763	
