# Supplementary material for: The major β-catenin/E-cadherin junctional binding site is a primary molecular mechano-transductor of differentiation in vivo
Source: eLife. 2018 Jul 19;7:e33381. doi: 10.7554/eLife.33381 (PMC6053302; doi:10.7554/eLife.33381)
Supplement: Figure 4—source data 1. [file elife-33381-fig4-data1.txt]

															
	WT					WT					WT				
	stage 5					stage 6 early					stage 6 late				
	meso	meso 	ecto	ecto		meso	meso	ecto	ecto		meso	meso	ecto	ecto	
	beta-cat	y654	beta-cat	y654		beta-cat	y654	beta-cat	y654		beta-cat	yy654	beta-cat	y654	
															
1	22947	17200	18333	15983	1	28993	26378	24563	21577	1	28221	25839	22123	22518	
2	24597	18206	20356	17786	2	18487	16227	18991	13472	2	10570	8889	9196	7084	
3	17444	12930	16025	13810	3	10533	8629	12510	7879	3	18360	15185	19541	13745	
4	25138	16724	22410	16476	4	11830	9342	9674	8624	4	18614	17649	20304	14327	
5	25045	17570	20822	17350	5	30793	27907	26024	21596	5	22364	19998	20406	16158	
															
															
															
	Src42aRNAi					Src42aRNAi					Src42aRNAi				
	stage 5					stage 6 early					stage 6 late				
	meso	meso	ecto	ecto		meso	meso	ecto	ecto		meso	meso	ecto	ecto	
	beta-cat	y654	beta-cat	y654		beta-cat	y654	beta-cat	y654		beta-cat	y654	beta-cat	y654	
															
1	31884	14999	34053	16292	1	4456	3214	5112	2830	1	48739	36288	45661	29712	
2	4023	1831	3530	1965	2	21842	16546	23974	13406	2	46260	29042	44889	21966	
3	17981	8563	14402	9570	3	8051	5215	7379	3867	3	13476	9944	12186	7616	
4	8791	4519	9948	4245	4	16583	11022	16726	8472	4	29449	18805	31890	16362	
5	23627	10917	19740	10144	5	15190	9746	13333	7618	5	29424	20634	27101	16851	
