# Supplementary material for: The major β-catenin/E-cadherin junctional binding site is a primary molecular mechano-transductor of differentiation in vivo
Source: eLife. 2018 Jul 19;7:e33381. doi: 10.7554/eLife.33381 (PMC6053302; doi:10.7554/eLife.33381)
Supplement: Transparent reporting form [file elife-33381-transrepform.docx]

***eLife’s* transparent reporting form**

We encourage authors to provide detailed information *within their submission* to facilitate the interpretation and replication of experiments. Authors can upload supporting documentation to indicate the use of appropriate reporting guidelines for health-related research (see [EQUATOR Network](http://www.equator-network.org/%20)), life science research (see the [BioSharing Information Resource](https://biosharing.org/)), or the [ARRIVE guidelines](http://www.plosbiology.org/article/info:doi/10.1371/journal.pbio.1000412) for reporting work involving animal research. Where applicable, authors should refer to any relevant reporting standards documents in this form.

If you have any questions, please consult our Journal Policies and/or contact us: [editorial@elifesciences.org](mailto:editorial@elifesciences.org).

**Sample-size estimation**

- You should state whether an appropriate sample size was computed when the study was being designed
- You should state the statistical method of sample size computation and any required assumptions
- If no explicit power analysis was used, you should describe how you decided what sample (replicate) size (number) to use

Please outline where this information can be found within the submission (e.g., sections or figure legends), or explain why this information doesn’t apply to your submission:

“A sample is one embryo or simulation, and one measurement is associated to one embryo or one simulation. N is the number of embryos or simulations by Figure, so N is the number of time the experiment was replicated both biologically and technically. Experiments and simulations are realized one by one on single embryos and single molecular complexes: the N sample size was thus designed *a posteriori* once the p-value showed statistically significant results enough, relative to the difficulty and time consuming of individual experiments and simulations. Statistics are based on the non parametric Mann-Whitney test, and the t-student test when allowed by the Shapiro-Wilk’s normality test two-sided. P values for comparing two curves were calculated following reference^71^ with Rstudio”.

This information can be found p25 in the statistics paragraph at the end of the Method section.

**Replicates**

- You should report how often each experiment was performed
- You should include a definition of biological versus technical replication
- The data obtained should be provided and sufficient information should be provided to indicate the number of independent biological and/or technical replicates
- If you encountered any outliers, you should describe how these were handled
- Criteria for exclusion/inclusion of data should be clearly stated
- High-throughput sequence data should be uploaded before submission, with a private link for reviewers provided (these are available from both GEO and ArrayExpress)

Please outline where this information can be found within the submission (e.g., sections or figure legends), or explain why this information doesn’t apply to your submission:

“A sample is one embryo or simulation, and one measurement is associated to one embryo or one simulation. N is the number of embryos or simulations by Figure, so N is the number of time the experiment was replicated both biologically and technically.” This information can be found p25 in the statistics paragraph at the end of the Method section.

N is given in all legend Figures or inside Figures.

**Statistical reporting**

- Statistical analysis methods should be described and justified
- Raw data should be presented in figures whenever informative to do so (typically when N per group is less than 10)
- For each experiment, you should identify the statistical tests used, exact values of N, definitions of center, methods of multiple test correction, and dispersion and precision measures (e.g., mean, median, SD, SEM, confidence intervals; and, for the major substantive results, a measure of effect size (e.g., Pearson's r, Cohen's d)
- Report exact p-values wherever possible alongside the summary statistics and 95% confidence intervals. These should be reported for all key questions and not only when the p-value is less than 0.05.

Please outline where this information can be found within the submission (e.g., sections or figure legends), or explain why this information doesn’t apply to your submission:

Raw data are shown as individual dots in all experiments, except in Fig.4c to make the Figure lighter (but can be added under request).

“Statistics are based on the non parametric Mann-Whitney test, and the t-student test when allowed by the Shapiro-Wilk’s normality test two-sided. P values for comparing two curves were calculated following reference^71^ with Rstudio.” This information can be found p25 in the statistics paragraph at the end of the Method section.

The exact statistical tests used are described in any Figure legend with all associated parameters.

Exact p-values are given in each case.

(For large datasets, or papers with a very large number of statistical tests, you may upload a single table file with tests, Ns, etc., with reference to sections in the manuscript.)

**Group allocation**

- Indicate how samples were allocated into experimental groups (in the case of clinical studies, please specify allocation to treatment method); if randomization was used, please also state if restricted randomization was applied
- Indicate if masking was used during group allocation, data collection and/or data analysis

Please outline where this information can be found within the submission (e.g., sections or figure legends), or explain why this information doesn’t apply to your submission:

Quantitative analysis was systematically done by automatic microscope image analysis or simulation dedicated algorithms, within the exact same conditions for all control/perturbed sample data in series, independently of the unmasked group allocation done by the investigator.

This information can be found p25 in the statistics paragraph at the end of the Method section.

**Additional data files (“source data”)**

- We encourage you to upload relevant additional data files, such as numerical data that are represented as a graph in a figure, or as a summary table
- Where provided, these should be in the most useful format, and they can be uploaded as “Source data” files linked to a main figure or table
- Include model definition files including the full list of parameters used
- Include code used for data analysis (e.g., R, MatLab)
- Avoid stating that data files are “available upon request”

Please indicate the figures or tables for which source data files have been provided:

Figure 1c,d

Figure 1-supplementary Figure 2b

Figure 2c

Figure 3d

Figure 4c,d,e

Figure 5d

Figure 2-supplementary figure 2b
